# A statistical approach to correct X-ray response non-uniformity in microstrip detectors for high-accuracy and high-resolution total-scattering measurements

**DOI:** 10.1107/S1600577519002145

**Published:** 2019-04-05

**Authors:** Kenichi Kato, Yoshihito Tanaka, Miho Yamauchi, Koji Ohara, Takaki Hatsui

**Affiliations:** a RIKEN SPring-8 Center, 1-1-1 Kouto, Sayo-cho, Sayo-gun, Hyogo 679-5148, Japan; b JST, PRESTO, 4-1-8 Honcho, Kawaguchi, Saitama 332-0012, Japan; c Graduate School of Material Science, University of Hyogo, 3-2-1 Kouto, Kamigori-cho, Ako-gun, Hyogo 678-1297, Japan; d International Institute for Carbon-Neutral Energy Research (WPI-I2CNER), Kyushu University, 744 Moto-oka, Nishi-ku, Fukuoka 819-0395, Japan; e Department of Chemistry, Faculty of Science, Kyushu University, 744 Moto-oka, Nishi-ku, Fukuoka 819-0395, Japan; f Research and Utilization Division, Japan Synchrotron Radiation Research Institute (JASRI, SPring-8), 1-1-1 Kouto, Sayo-cho, Sayo-gun, Hyogo 679-5198, Japan

**Keywords:** total scattering, pair distribution functions, microstrip detectors, flat-field calibration, Poisson noise

## Abstract

An unbiased approach to correct X-ray response non-uniformity in microstrip detectors has been developed based on the statistical estimation that the scattering intensity at a fixed angle from an object is expected to be constant within the Poisson noise. Raw scattering data of SiO_2_ glass measured by a microstrip detector module was found to show an accuracy of 12σ_PN_ at an intensity of 10^6^ photons, where σ_PN_ is the standard deviation according to the Poisson noise. The conventional flat-field calibration has failed in correcting the data, whereas the alternative approach used in this article successfully improved the accuracy from 12σ_PN_ to 2σ_PN_. This approach was applied to total-scattering data measured by a gapless 15-modular detector system. The quality of the data is evaluated in terms of the Bragg reflections of Si powder, the diffuse scattering of SiO_2_ glass, and the atomic pair distribution function of TiO_2_ nanoparticles and Ni powder.

## Abstract 2

A statistical approach to correct X-ray response non-uniformity in microstrip detectors was developed and applied to a detector system, which enabled high-accuracy and high-resolution total scattering measurements in a wide range of scattering vector.

## 1. Introduction   

The reliability of results obtained by X-ray structural analysis depends on accuracy and precision in diffraction and scattering intensity from a sample. In particular, this applies to atomic pair distribution function (PDF) analysis of total-scattering data since real-space structure information can be obtained via no structural model. The total-scattering PDF method has been employed to characterize the local structure of crystalline materials as well as amorphous materials (Egami & Billinge, 2012 ▸). In contrast, it is widely recognized that Rietveld analysis of Bragg diffraction data provides fundamental understanding on the average structure of crystalline materials. In brief, the two methods have been considered to be complementary to each other in crystal structural analysis.

The term ‘total scattering’ is derived from treating Bragg and diffuse scattering on an equal basis. In other words, total-scattering experiments are considered to be on the extension of conventional powder diffraction experiments. Nevertheless, data measurements for PDF analysis have been conducted independently of measurements for Rietveld analysis. In general, PDF analysis requires total-scattering data in a wide range of scattering vector, **Q**, which has a magnitude |**Q**| = *Q* = 4π sin(2θ/2)/λ, where λ is the wavelength of scattered X-rays and 2θ is the scattering angle. In contrast, Rietveld analysis needs high-*Q*-resolution data. The problem is how to obtain total-scattering data with both high *Q* resolution and wide *Q* space, which are considered to be in a trade-off relationship. Such ideal data would facilitate multi-scale PDF analysis. Among other local structure probes such as extended X-ray absorption fine structure, the total-scattering PDF method has a potential for investigating heterogeneous phenomena in functional nanomaterials (Skrobas *et al.*, 2017 ▸).

The rapid-acquisition PDF (RA-PDF) method (Chupas *et al.*, 2003 ▸, 2007 ▸), which is typical of total-scattering beamlines at synchrotron facilities, can collect total-scattering data with the maximum *Q* value (*Q*_max_) above 30 Å^−1^ by a combination of high-energy X-rays and a large area detector. The *Q* resolution (∼10^−2^ Å^−1^) is moderate for diffuse scattering but is insufficient for Bragg reflections because of the use of energies of around 100 keV, a short sample-to-detector distance and a low-resolution detector. As a consequence, the PDF obtained by RA-PDF undergoes significant damping at longer inter­atomic distances (Saleta *et al.*, 2017 ▸). On the other hand, point detectors such as scintillation counters allow high-resolution measurements by setting analyzer crystals (Saleta *et al.*, 2017 ▸). It is not, however, technically feasible to cover a wide range of *Q* at the same time even by using multiple point detectors. For that reason, such a scanning method cannot be applied to *in situ* experiments under non-ambient conditions, where a scattering pattern varies every moment.

A number of powder diffraction beamlines (Haverkamp & Wallwork, 2009 ▸; Bergamaschi *et al.*, 2010 ▸; Thompson *et al.*, 2011 ▸; Saleta *et al.*, 2017 ▸) at synchrotron facilities have employed an array of microstrip detectors such as MYTHEN (DECTRIS Ltd, Baden-Daettwil, Switzerland) (Schmitt *et al.*, 2003 ▸). In addition to powder diffraction data, such a MYTHEN detector array can measure high-*Q*-resolution (∼10^−4^ Å^−1^) total-scattering data in a wide range of *Q* (20–30 Å^−1^) even by using moderate energies of around 20–30 keV. Indeed, the detector array has been tested for the PDF method (Haverkamp & Wallwork, 2009 ▸; Saleta *et al.*, 2017 ▸). However, multi-scale PDF analysis at interatomic distances above 100 Å has never been conducted, which is likely to be because of X-ray response non-uniformity (XRNU) in MYTHEN. In X-ray detectors, XRNU has been widely recognized as one of the most critical problems for structural analysis. To correct XRNU, the conventional flat-field calibration has been applied to MYTHEN detector arrays (Bergamaschi *et al.*, 2010 ▸; Thompson *et al.*, 2011 ▸). In this calibration, each module is illuminated with a roughly flat X-ray pattern with a specific energy, which is obtained by scanning a stable beam. The XRNU on each channel is calibrated based on the difference between raw data and the completely flat pattern. The calibration can correct the relatively large XRNU as observed at both ends of channels (Thompson *et al.*, 2011 ▸) but has failed in correcting the small XRNU found at intensities above 10^4^ photons because of an approximation to the completely flat pattern, which is impossible to obtain.

Here we propose an approach to correct XRNU in microstrip detectors without using any approximation, which could be a replacement for the conventional flat-field calibration. The alternative approach is based on the statistical estimation that the scattering intensity at a fixed angle from an object is expected to be constant within the Poisson noise. The improvements in total-scattering data measured by a MYTHEN-modular detector system are discussed from the point of view of two figures of merit for X-ray detectors and PDFs.

## 2. Instruments and methods   

### 2.1. X-ray total-scattering detector   

#### 2.1.1. Detector module   

Among other X-ray detectors, a photon-counting microstrip detector, MYTHEN, was selected as a promising candidate for high-accuracy and high-resolution total-scattering measurements. The detector module can meet the demands for spatial resolution and dynamic range in such measurements. An interval between two adjacent strips is 50 µm, which corresponds to the spatial resolution of the module. The counter has a dynamic range of the order of 10^7^. In addition, such a photon-counting system can separate signal from noise by setting the optimal threshold energy for each incident energy to exclude readout and dark noise. The module is composed of an electronic board and a Si sensor, the dimensions of which are 64 mm by 8 mm. The sensor with a thickness of 1 mm was employed to improve quantum efficiency at high energies.

#### 2.1.2. Detector system   

**Figure 1:**
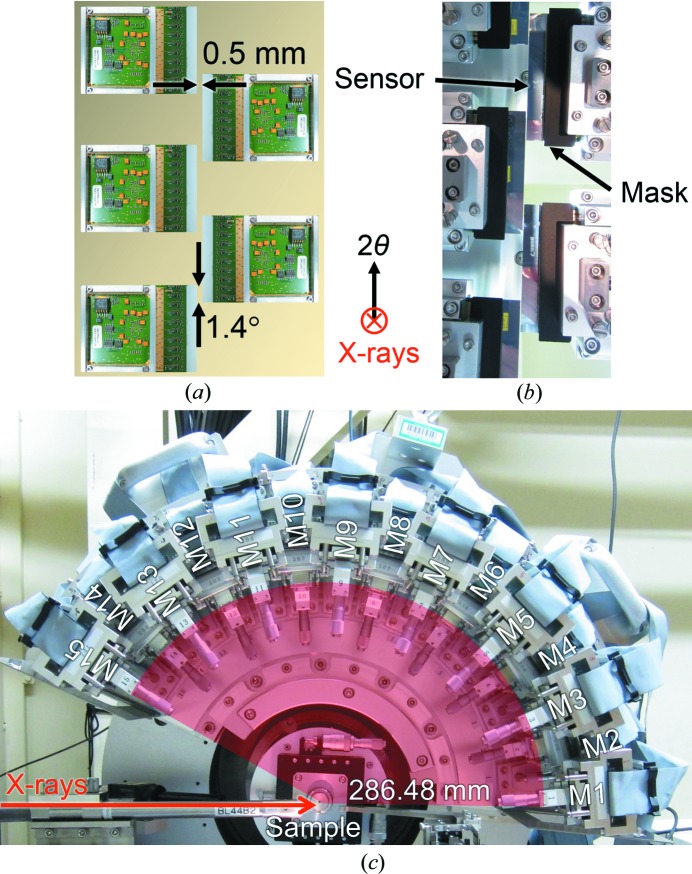
The gapless MYTHEN-modular detector system. (*a*) A schematic view of the gapless arrangement with five modules. (*b*) A corresponding photograph of (*a*), where masks can be seen in front of the sensors. (*c*) A photograph of the 15-modular system, which has been installed on the Debye–Scherrer camera at BL44B2 of SPring-8. Note that the photographs in (*b*) and (*c*) were taken without housing.

To measure total-scattering data in a wide range of *Q* in parallel, 15 modules without housing were assembled to build a detector system, which was installed on the Debye–Scherrer camera of the RIKEN Materials Science beamline BL44B2 (Kato *et al.*, 2010 ▸; Kato & Tanaka, 2016 ▸) of SPring-8 (Fig. 1 ▸). These modules were arranged in a pseudo curve on the 2θ stage of the camera. The distance between the sample position and the sensor surface was fixed at 286.48 (1) mm so that the angular resolution is ∼0.01° in 2θ. The detector system was characterized by its gapless arrangement in the vertical direction as shown in Fig. 1 ▸(*a*). Adjacent modules overlapped each other by 140 channels, which correspond to 1.4° in 2θ. The overlap can detect the identical Bragg reflection by two adjacent modules. Comparing these two intensities leads to the evaluation of granularity and preferred orientation of powder samples. Thanks to the arrangement, total-scattering data in the vertical direction can be collected up to 153° in 2θ simultaneously, which corresponds to 30 Å^−1^ in *Q*_max_ in the case of 30 keV in incident energy. In contrast, there is a 0.5 mm gap in the horizontal direction between adjacent modules. Including the insensitive edge (1 mm) of the sensor, the width of ±1.25 mm from the horizontal center is lost in data. The full width of the sensor is 8 mm. The aperture for scattered X-rays can be adjusted using a brazen mask of 2 mm thickness in front of the sensor [Fig. 1 ▸(*b*)]. The mask edge was aligned along the sensor edge within an accuracy of 0.3% in aperture. The XRNU observed in the detector system is affected by the misalignment of the mask to the sensor and the roughness of the mask edge. Taking into account the umbrella effect at lower angles and relatively low statistics at higher angles, the apertures of the 15 modules were fixed for the present experiments as follows: 0.50 mm for module 1 (M1), 0.75 mm for M2, 1.00 mm for M3, 1.25 mm for M4, 1.50 mm for M5, 1.75 mm for M6, 2.00 mm for M7, 2.25 mm for M8, 2.50 mm for M9, 2.75 mm for M10, 3.00 mm for M11, 3.25 mm for M12, 3.50 mm for M13, 3.75 mm for M14 and 4.00 mm for M15 [Fig. 1 ▸(*c*)]. The detector system is controlled using a measurement program written by the graphical programming software *LabVIEW* (National Instruments Corporation, Austin, TX, USA) via the detector control system DCS24 (DECTRIS Ltd).

### 2.2. Approach to correct XRNU   

#### 2.2.1. Concept   

**Figure 2:**
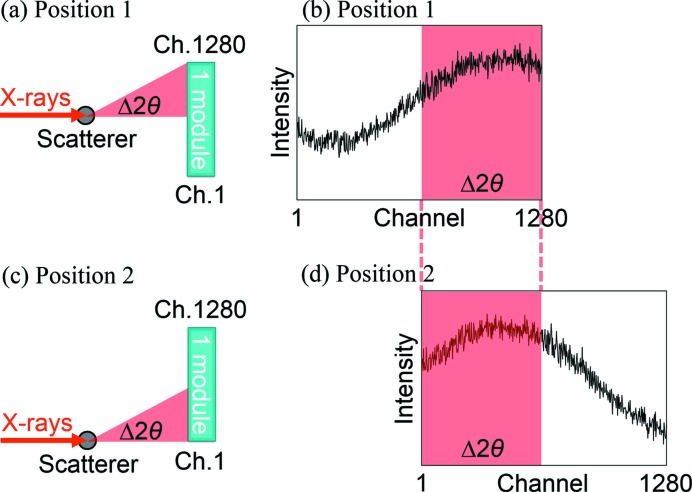
Schematic drawing of the statistical approach at two module positions using one module. (*a*) The setup and (*b*) the scattering data at module position 1. Likewise, (*c*) the setup and (*d*) the scattering data at module position 2. Without any systematic error, the intensity distribution at Δ2θ in (*b*) is expected to be consistent with that at Δ2θ in (*d*) within the Poisson noise.

Our approach to correct XRNU is based on the statistical estimation that the scattering intensity at a fixed angle from an object is expected to be constant within the Poisson noise. The conventional flat-field calibration employs an approximation to the perfectly uniform illumination of X-rays, which is impossible to obtain. In contrast, the alternative approach is characterized by no need for such an approximation. For example, the scattering intensity distribution at a fixed angle range Δ2θ measured by module position 1 [Figs. 2 ▸(*a*) and 2 ▸(*b*)] should be in agreement with that at Δ2θ by position 2 [Figs. 2 ▸(*c*) and 2 ▸(*d*)] within the Poisson noise. In practice, however, these distributions are in disagreement because of various systematic errors including XRNU. The approach is expected to correct XRNU based on the difference between the two distributions at Δ2θ measured by different channels. Therefore, there should be no systematic difference in XRNU between compared channels. In other words, the key to success in correction is randomness in XRNU between channels. From this fact, the module should not be partitioned every 128 channels, which corresponds to an architectural unit of the readout chip.

#### 2.2.2. Divided-accumulation technique   

The statistical approach requires that the scattering intensity at a fixed angle from an object should not exceed 0.1% in relative fluctuation, which corresponds to the Poisson noise at 10^6^ photons. The fluctuation of the ring current at SPring-8 is controlled below 0.05% by the top-up injection in any electron bunch mode. We, nevertheless, found it difficult to maintain the 0.1% fluctuation because of the thermal instability of optical and measuring systems. To average out fluctuations in scattering intensity, we have employed a divided-accumulation technique, where two measurements at module positions 1 and 2 [Figs. 2 ▸(*a*) and 2 ▸(*c*)] are repeated until the total intensity at each position reaches the order of 10^6^ photons. The important point is that measurement time at each position is fixed so that the cycle of measurements is sufficiently shorter than that of fluctuations in scattering intensity. After a series of measurements, multiple data at the same position are summed up to yield a two-data set. The technique can be applied to detectors without readout noise such as photon-counting-type detectors since a large amount of data is accumulated.

#### 2.2.3. Scattering object and conditions   

A SiO_2_ glass rod with a diameter of 3.5 mm was used as the scattering object for the approach. Among others, the object can give rise to high and reproducible scattering intensity in a wide range of *Q*. The wavelength of incident X-rays was set at 0.45 Å, taking into account the quantum efficiency of the 1 mm-Si sensor. The threshold energy in the module was set at half of the incident energy to avoid double counts. The wavelength was calibrated by the use of the *K* absorption edge of In with a precision of 10^−4^ Å. The horizontal and vertical sizes of the incident slit were 3 mm and 1 mm, respectively, which is smaller than the object size to reduce the influence of fluctuations in beam position.

#### 2.2.4. Single-step process   

In Fig. 2 ▸, only two module positions are illustrated for simplicity. Based on the concept, in practice, measurements were conducted at 160 positions, which is referred to as the single-step process. One module has 1280 channels, which are indexed from 1 to 1280 in order. The 1280 channels were partitioned into every eight channels, which corresponds to about 0.08° in 2θ in the sample-to-detector distance. The partition can be expressed in terms of a 8 × 160 matrix,
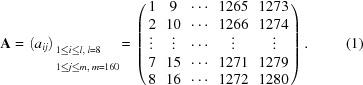
The process was combined with the divided-accumulation technique described in Section 2.2.2, in which case the following sequence of measurements was repeated until the total intensity at a fixed module angle 2θ_M_ reached the order of 10^6^ photons:

(1) One-minute measurement at 2θ_M_,

(2) One-minute measurement at 2θ_M_ + 0.08°,

(3) One-minute measurement at 2θ_M_ + 0.08° × 2,


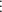


(160) One-minute measurement at 2θ_M_ + 0.08° × 159.

Multiple one-minute data measured at the same module angle were summed up to yield a 160-data set. Correction factors *c*_ss_ for individual channels based on the single-step were calculated by taking an average of the intensity measured by 160 different channels at a fixed scattering angle 2θ according to the following expression,
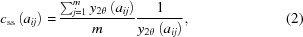
where 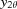 is the scattering intensity at 2θ for each channel. Note that 32 channels at both ends (channels 1–16 and 1265–1280) were eliminated from the calculation since their intensities were unreliable because of the unpredictable response.

#### 2.2.5. Multi-step process   

The number of measurements in the single-step process is 160, which takes several-ten hours to obtain intensity of the order of 10^6^ photons. To reduce the total time maintaining the correction effects, a multi-step process based on the single-step was developed. The process consists of five steps. The number of measurements in the multi-step is 24, which is 15% of that in the single-step.

For the first-step calculation, 1280 channels were partitioned into every 80 channels, which corresponds to 0.8° in 2θ, with a 80 × 16 matrix,
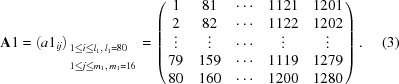
In the same way as the single-step, the following sequence was repeated:

(1) Three-minute measurement at 2θ_M1_,

(2) Three-minute measurement at 2θ_M1_ + 0.8°,

(3) Three-minute measurement at 2θ_M1_ + 0.8° × 2,


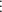


(16) Three-minute measurement at 2θ_M1_ + 0.8° × 15.

Using the 16-data set, correction factors *c*1 were calculated by the following expression,
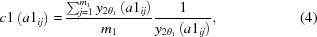
where 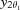 is the scattering intensity at a fixed scattering angle 2θ_1_ for each channel. Note that 32 channels at both ends (channels 1–16 and 1265–1280) were eliminated from the calculation as in the single-step process.

For the second-step calculation, 1280 channels were repartitioned into every 40 channels, which corresponds to 0.4° in 2θ, with a three-dimensional 40 × 2 × 16 matrix,
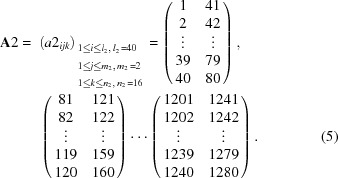
Similarly, the following sequence was repeated:

(1) Three-minute measurement at 2θ_M2_,

(2) Three-minute measurement at 2θ_M2_ + 0.4°.

Using the two-data set, correction factors *c*2 were calculated by the following expression,
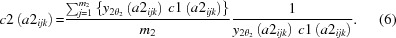
For the third-step calculation, 1280 channels were repartitioned into every 20 channels, which corresponds to 0.2° in 2θ, with a three-dimensional 20 × 2 × 32 matrix,
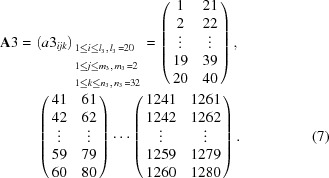
Similarly, the following sequence was repeated:

(1) Three-minute measurement at 2θ_M3_,

(2) Three-minute measurement at 2θ_M3_ + 0.2°.

Using the two-data set, correction factors *c*3 were calculated by the following expression,
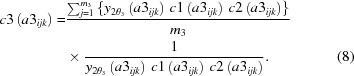
For the fourth-step calculation, 1280 channels were repartitioned into every 10 channels, which corresponds to 0.1° in 2θ, with a three-dimensional 10 × 2 × 64 matrix,
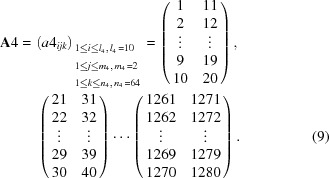
Similarly, the following sequence was repeated:

(1) Three-minute measurement at 2θ_M4_,

(2) Three-minute measurement at 2θ_M4_ + 0.1°.

Using the two-data set, correction factors *c*4 were calculated by the following expression,
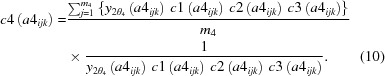
For the fifth-step calculation, 1280 channels were repartitioned into every five channels, which corresponds to 0.05° in 2θ, with a three-dimensional 5 × 2 × 128 matrix,
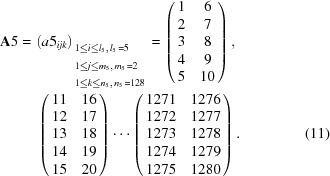
Similarly, the following sequence was repeated:

(1) Three-minute measurement at 2θ_M5_,

(2) Three-minute measurement at 2θ_M5_ + 0.05°.

Using the two-data set, correction factors *c*5 were calculated by the following expression,
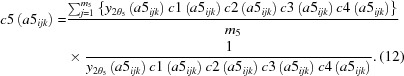
The final correction factors *c*_ms_ for individual channels based on the multi-step process is expressed by
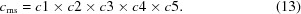



#### 2.2.6. Application to the detector system   

In Sections 2.2.4 and 2.2.5, one module is considered for simplicity. In practice, however, the multi-step process combined with the divided-accumulation technique was applied to the 15-modular system with 1280 × 15 = 19200 channels. Our approach to correct XRNU has another advantage in that the number of measurements for correction factors is not influenced by the number of modules since the SiO_2_ glass scattering is continuously distributed in a wide range of *Q*. In brief, measurements for correction factors of 15 modules can be carried out in parallel.

### 2.3. Figure of merit for X-ray detectors   

To examine the quality of scattering data measured by X-ray detectors, we have defined total fractional uncertainty (TFU) as a figure of merit,
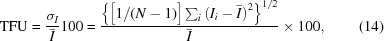
where 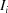 is the scattering intensity of channel *i*, 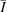 is the average of 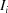, 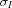 is the standard deviation of 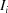 and *N* is the number of channels.

In addition, another figure of merit has been introduced as the ratio of 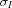 to the standard deviation based on the Poisson noise 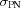 (Poisson noise ratio or PNR),
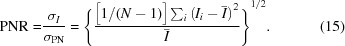



### 2.4. Sample measurements and PDF analysis   

To consider the quality of corrected data from various aspects, four different kinds of samples were measured by the detector system using the incident X-ray wavelength of 0.45 Å, which is the same as that in measurements for correction factors. The threshold energy in the detector system was set at half of the incident energy, which is the same as that in measurements for correction factors. The four samples were: a SiO_2_ glass rod, 3.5 mm in diameter; Si powder (Standard Reference Material 640d; NIST, Gaithersburg, MD, USA), filled into a Lindemann-glass capillary (No. 14; Hilgenberg GmbH, Malsfeld, Germany), 0.3 mm in diameter; anatase-type TiO_2_ powder, ∼10 nm in diameter, filled into a Lindemann-glass capillary, 0.3 mm in diameter; and Ni powder (NI-314010; The Nilaco Corporation, Tokyo, Japan), filled into a Lindemann-glass capillary, 0.1 mm in diameter. In addition, empty capillaries were measured under the same experimental conditions for PDF analysis. Intensity data indexed from channels 1 to 19200 were converted into 2θ and *Q* by the calibration using a line-position and -shape standard for powder diffraction, LaB_6_ powder (Standard Reference Material 660b; NIST). PDF analysis was performed using the *xPDFsuite* software (Yang *et al.*, 2014 ▸). For the analysis, *Q*_max_ and the minimum *Q* value were 27 Å^−1^ and 0.9 Å^−1^, respectively.

It is known that counting efficiency is influenced by counting rate in photon-counting-type detectors, yielding XRNU between channels in microstrip detectors. In the present study, the photon arrival rate from samples was set at between 10^2^ and 10^3^ photons s^−1^, which is much lower than the maximum counting rate (10^5^ photons s^−1^). This means that counting loss becomes insignificant.

## 3. Results   

### 3.1. Correction factors and their application   

**Figure 3:**
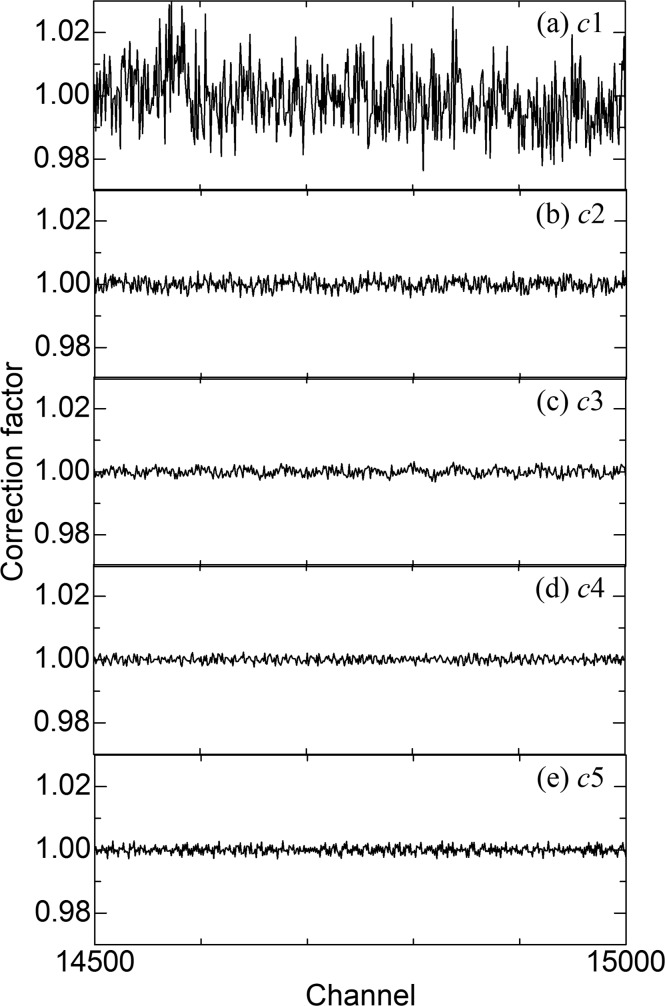
Correction factors from channels 14500 to 15000 at each step in the multi-step process: (*a*) the first-step factors *c*1 in equation (4), (*b*) the second-step factors *c*2 in equation (6), (*c*) the third-step factors *c*3 in equation (8), (*d*) the fourth-step factors *c*4 in equation (10) and (*e*) the fifth-step factors *c*5 in equation (12).

The application of the multi-step process to the 15-modular system yielded correction factors from channels 1 to 19200. Correction factors from channels 14500 to 15000 at each step are shown in Fig. 3 ▸ among these factors. The first-step factors *c*1 fluctuate between 0.98 and 1.03 [Fig. 3 ▸(*a*)]. The fluctuations of the second-step factors *c*2 are much smaller than those of the first-step [Fig. 3 ▸(*b*)]. Step by step, the fluctuations of the correction factors decrease, indicating that the multi-step functions effectively [Figs. 3 ▸(*c*), 3 ▸(*d*) and 3 ▸(*e*)].

**Figure 4:**
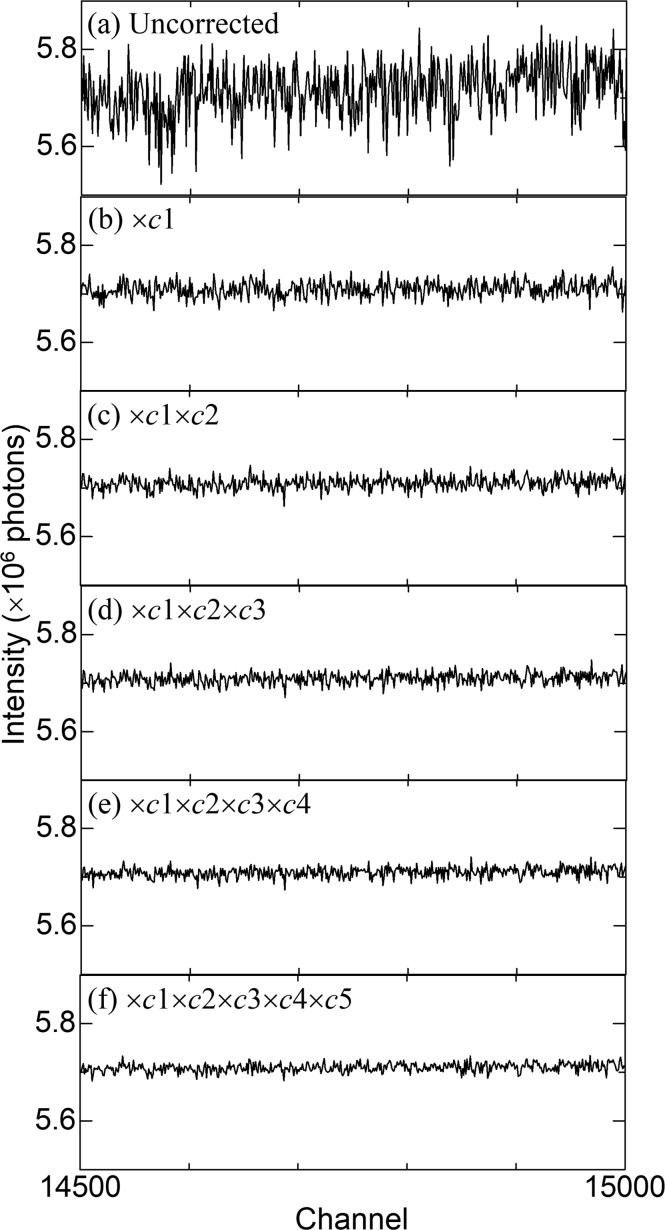
The scattering data of SiO_2_ glass from channels 14500 to 15000 at each step in the multi-step process: (*a*) uncorrected; (*b*) multiplied by *c*1 in equation (4); (*c*) multiplied by *c*1 in equation (4) and *c*2 in equation (6); (*d*) multiplied by *c*1 in equation (4), *c*2 in equation (6) and *c*3 in equation (8); (*e*) multiplied by *c*1 in equation (4), *c*2 in equation (6), *c*3 in equation (8) and *c*4 in equation (10); and (*f*) multiplied by *c*_ms_ in equation (13).

The scattering data of SiO_2_ glass were measured by the detector system with 19200 channels and then corrected by correction factors at each step in the multi-step process. Fig. 4 ▸ only shows the data measured from channels 14500 and 15000, which corresponds to ∼123.6°–128.6° in 2θ. The average intensity of the range is ∼5.7 × 10^6^ photons. According to the Poisson statistics, the standard deviation of the range is expected to be the square root of 5.7 × 10^6^ (= 2387). However, the uncorrected data deviate sharply from the expected values [Fig. 4 ▸(*a*)]. The data corrected by the first-step factors *c*1 indicate that the deviations decrease [Fig. 4 ▸(*b*)]. Step by step, the deviations show a gradual decrease [Figs. 4 ▸(*c*), 4 ▸(*d*), 4 ▸(*e*) and 4 ▸(*f*)].

**Figure 5:**
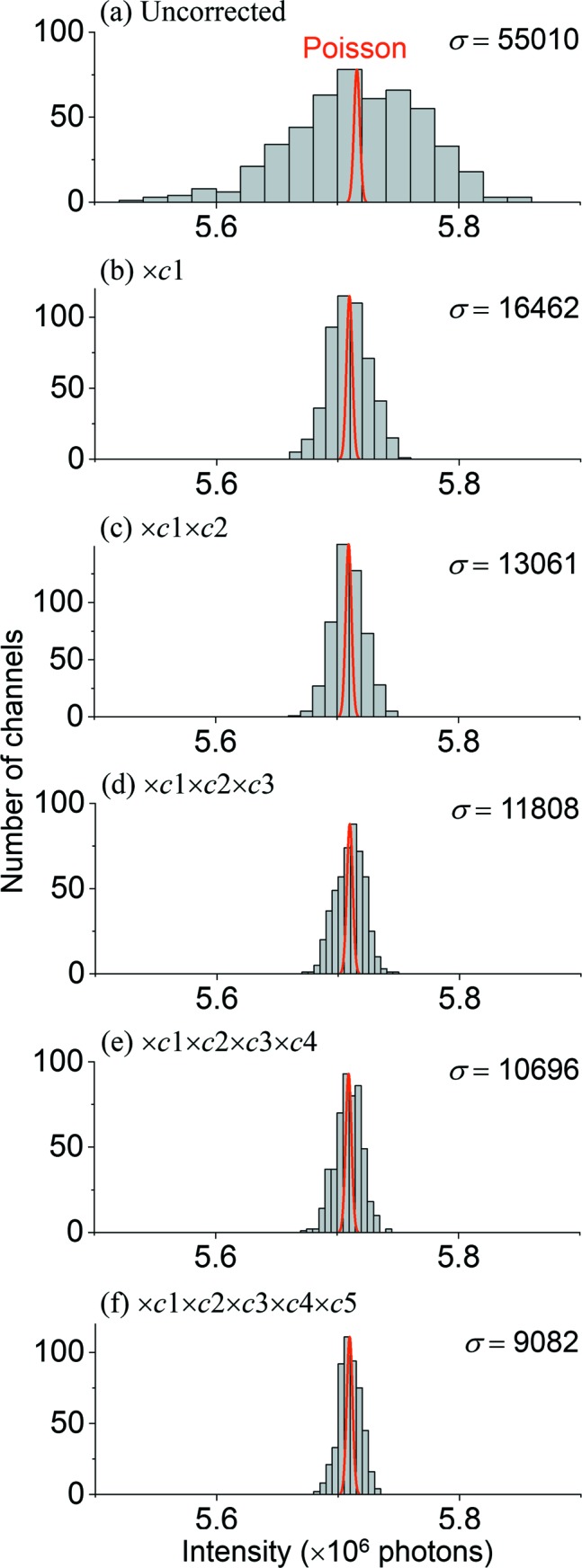
Intensity distribution of SiO_2_ glass from channels 14500 and 15000 at each step. (*a*) The uncorrected, (*b*) first-step, (*c*) second-step, (*d*) third-step, (*e*) fourth-step and (*f*) fifth-step distributions. The Poisson distribution is also plotted for comparison.

Fig. 5 ▸ shows histograms of the scattering data of SiO_2_ glass from channels 14500 to 15000 at each step, which have the horizontal axis of intensity and the vertical axis of number of channels. The uncorrected data have a wide distribution with a standard deviation of 55010 photons [Fig. 5 ▸(*a*)]. The application of the first-step factors *c*1 reduces the deviation to 16462 photons [Fig. 5 ▸(*b*)]. Step by step, the distributions become sharp, indicating an approach to the Poisson distribution [Figs. 5 ▸(*c*), 5 ▸(*d*) and 5 ▸(*e*)]. The final standard deviation is 9082 photons at 5.7 × 10^6^ photons, which is reduced by a factor of six relative to that of the uncorrected data [Fig. 5 ▸(*f*)].

### 3.2. TFU and PNR   

**Figure 6:**
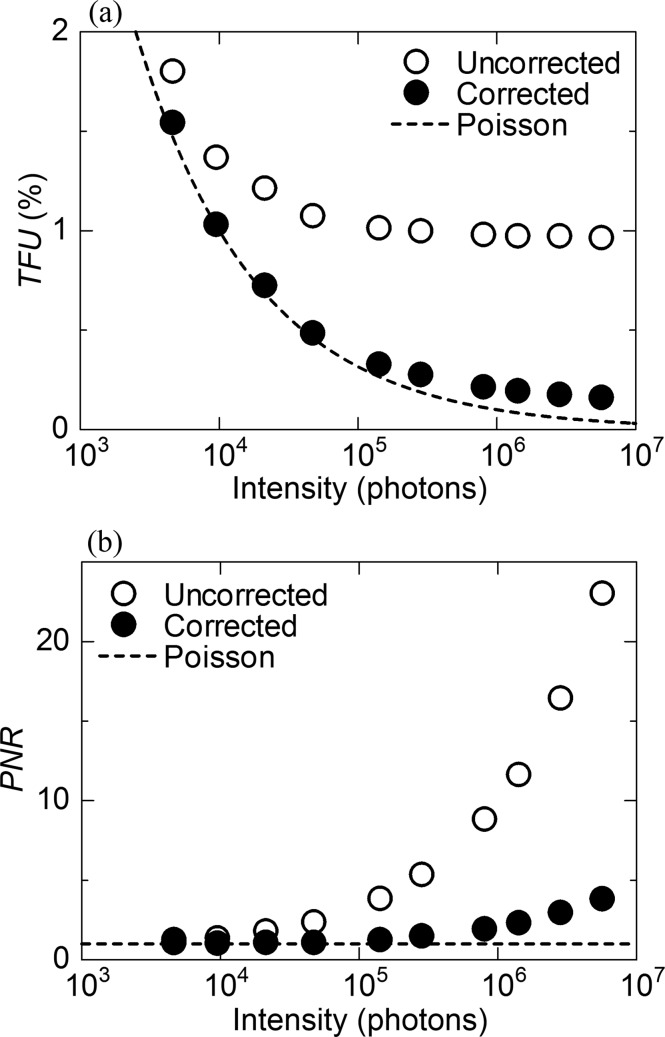
(*a*) TFU (*i*: 14500–15000, *N* = 501) and (*b*) PNR (*i*: 14500–15000, *N* = 501) of the uncorrected and corrected scattering data of SiO_2_ glass, as a function of average intensity. The Poisson curve is also plotted for comparison.

Fig. 6 ▸(*a*) shows the TFU (*i*: 14500–15000, *N* = 501) of the corrected and uncorrected scattering data of SiO_2_ glass as a function of average intensity, which is defined as a figure of merit for X-ray detectors in equation (14). To compare the systematic error such as XRNU with the statistical error, the fractional uncertainty curve based on the Poisson statistics was also plotted. In the uncorrected data, the deviations from the Poisson noise are significant at intensities above 10^4^ photons, reaching a plateau above 10^5^ photons. In contrast, the corrected data are found to be close to the Poisson noise even at intensities above 10^6^ photons.

Fig. 6 ▸(*b*) shows the PNR (*i*: 14500–15000, *N* = 501) of the corrected and uncorrected scattering data of SiO_2_ glass as a function of average intensity, which is defined as another figure of merit for X-ray detectors in equation (15). The PNR of the uncorrected data increases exponentially with increasing average intensity, resulting in 23.0 at 5.7 × 10^6^ photons. In contrast, the corrected data maintain the PNR of 2.3 even at 1.4 × 10^6^ photons, leading to 3.8 at 5.7 × 10^6^ photons.

### 3.3. Effects of the multi-step process and the mask   

**Figure 7:**
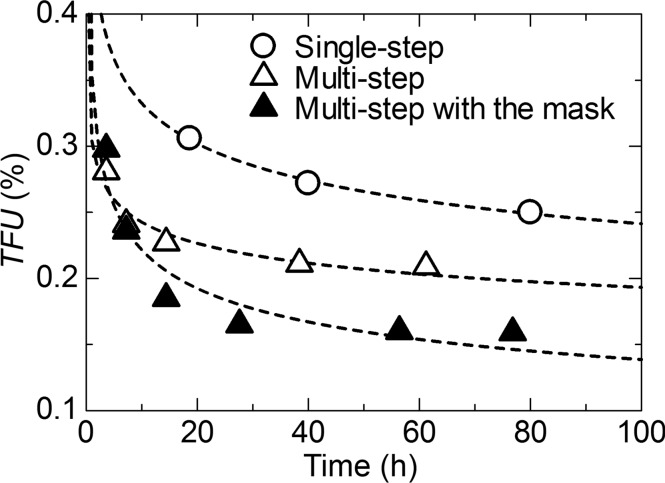
TFU (*i*: 14500–15000, *N* = 501) of the corrected scattering data of SiO_2_ glass with intensity of the order of 10^6^ photons, as a function of measurement time for correction factors. The single-step process without the mask and the multi-step with and without the mask are indicated.

To examine improvements in data quality by the multi-step process and the mask in front of the sensor, the TFU (*i*: 14500–15000, *N* = 501) in the SiO_2_ glass scattering data with the order of 10^6^ photons in intensity was plotted as a function of measurement time for correction factors in Fig. 7 ▸. The single-step without the mask shows a gradual decrease in TFU with increasing time, resulting in 0.25% at 80 h. On the other hand, the TFU in the multi-step without the mask reaches 0.25% within 8 h, which is an order of magnitude shorter than the single-step. The significant reduction in measurement time indicates that some multiplier effects work on data correction in the multi-step. Furthermore, the TFU in the multi-step is further improved by the use of the mask. The observations suggest that the mask is useful in reducing some noise, which might be caused by an exposure of scattered X-rays to the wire bonding. Notice that the TFU before the XRNU correction with the mask was comparable with that without the mask. The results indicate that the accuracy of the alignment is sufficient to discuss XRNU.

### 3.4. Improvements in total-scattering data   

Total-scattering measurements take into account both Bragg and diffuse scattering. This section describes the improvements in Bragg reflections of Si powder and diffuse scattering of SiO_2_ glass by our approach.

#### 3.4.1. Bragg reflections   

**Figure 8:**
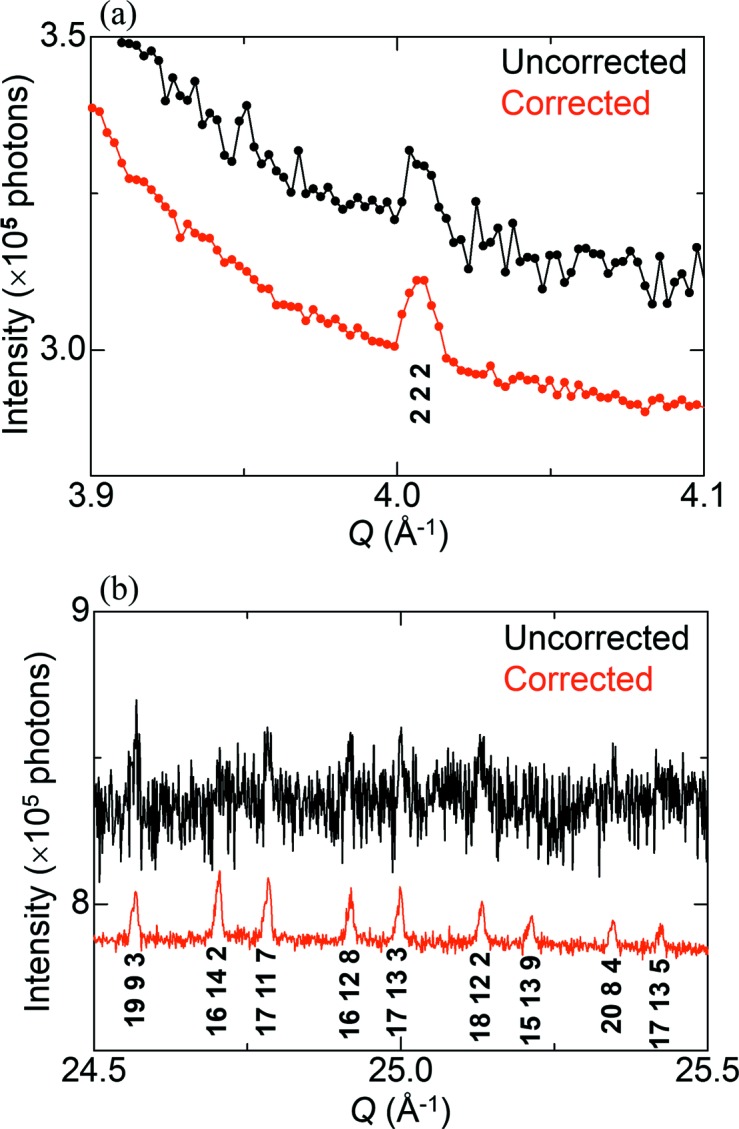
Part of Bragg reflections in the uncorrected and corrected data of Si powder. (*a*) The forbidden reflection 222 and (*b*) higher-order reflections. For clarity, the corrected data are shifted slightly lower. Note that each higher-order reflection is indicated by the primary index.

Si powder was measured to examine the improvements in Bragg reflections. Fig. 8 ▸(*a*) shows the forbidden reflection 222 in the uncorrected and corrected data, the intensity of which is three orders of magnitude lower than that of the primary reflection 111. The forbidden reflection is clearly observed in the corrected data as compared with the uncorrected data. Fig. 8 ▸(*b*) shows higher-order reflections around 25 Å^−1^ in *Q*, the intensity of which is four orders of magnitude lower than that of 111. These reflections in the uncorrected data are hidden under noise, whereas those in the corrected data appear at the expected peak positions.

#### 3.4.2. Diffuse scattering   

**Figure 9:**
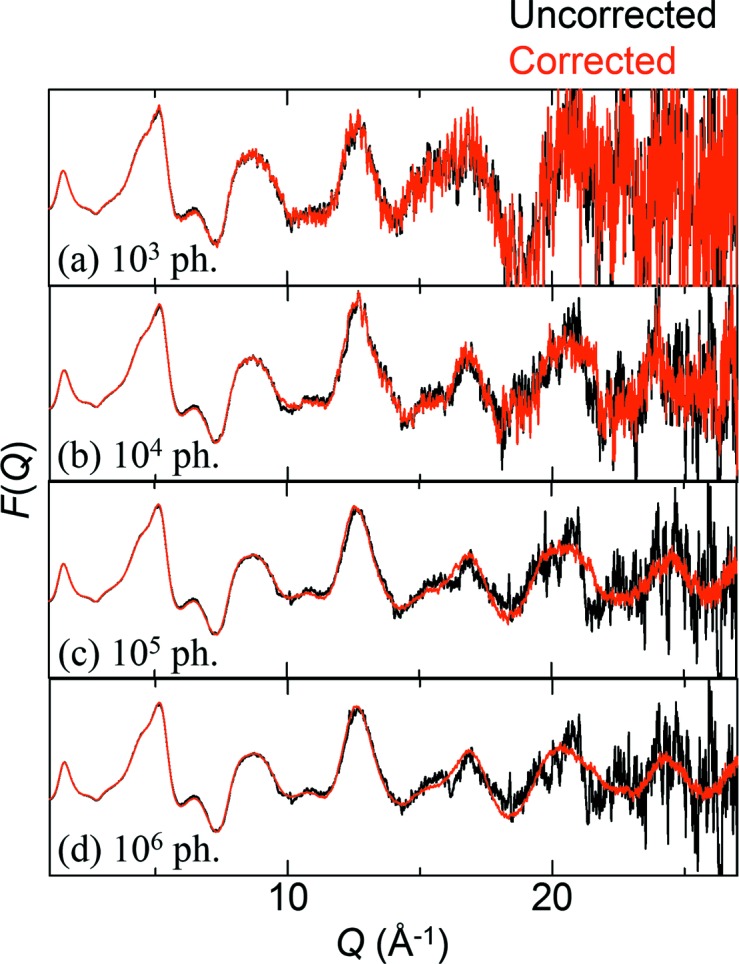
Reduced structure function *F*(*Q*) based on the uncorrected and corrected SiO_2_ glass data with intensities of the order of (*a*) 10^3^, (*b*) 10^4^, (*c*) 10^5^ and (*d*) 10^6^ photons. Note that the moving average (±10 channels) was conducted in the uncorrected and corrected data used for the *F*(*Q*) to improve statistics at high *Q*.

The scattering data of SiO_2_ glass were converted into the reduced structure function *F*(*Q*) = *Q*[*S*(*Q*) − 1] to examine the improvements in diffuse scattering. Fig. 9 ▸ shows the *F*(*Q*) obtained based on the uncorrected and corrected data as a function of scattering intensity (10^3^ to 10^6^ photons). The *F*(*Q*) based on the corrected and uncorrected data with 10^3^ photons is too noisy to identify diffuse scattering especially at *Q* above 20 Å^−1^, which is caused by insufficient statistics [Fig. 9 ▸(*a*)]. With increasing intensity, the uncorrected data undergo almost no change above 10^4^ photons, whereas the corrected data are found to be improved at high *Q* according to photon statistics [Figs. 9 ▸(*b*), 9 ▸(*c*) and 9 ▸(*d*)].

### 3.5. Improvements in PDFs   

The total-scattering PDF, which can be obtained without any structural models, is considered to be one of the figures of merit for X-ray detectors. This section describes the improvements by our approach in the PDF of two crystalline powders with different crystallite sizes.

#### 3.5.1. Nanometre crystal   

**Figure 10:**
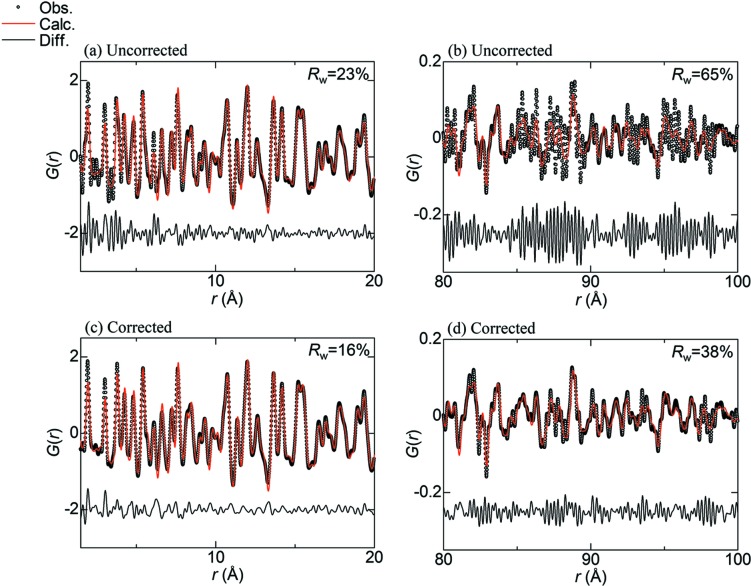
PDF *G*(*r*) of TiO_2_ nanoparticles fitted by the anatase-type structural model. Firstly, (*a*) and (*b*) show the results based on the uncorrected data in the range 1.5–20 Å and 80–100 Å in interatomic distance *r*, respectively. Secondly, (*c*) and (*d*) show the results based on the corrected data in the range 1.5–20 Å and 80–100 Å in *r*, respectively. Reliability factors *R*_w_ and the difference between observed and calculated PDFs are shown at the upper right and below in each figure, respectively.

The PDF analysis of anatase-type TiO_2_ nanoparticles was performed every 20 Å in interatomic distance. Fig. 10 ▸ shows the fitting results in the range 1.5–20 Å and 80–100 Å, the latter of which is close to the crystallite size. The reliability factors *R*_w_ based on the uncorrected data indicate 23% and 65% at the short and long ranges, respectively [Figs. 10 ▸(*a*) and 10 ▸(*b*)]. On the other hand, the *R*_w_ based on the corrected data are 16% and 38% at the short and long ranges, respectively [Figs. 10 ▸(*c*) and 10 ▸(*d*)]. The improvements in *R*_w_ indicate that the PDF from the corrected data is more reliable than that from the uncorrected data.

#### 3.5.2. Micrometre crystal   

**Figure 11:**
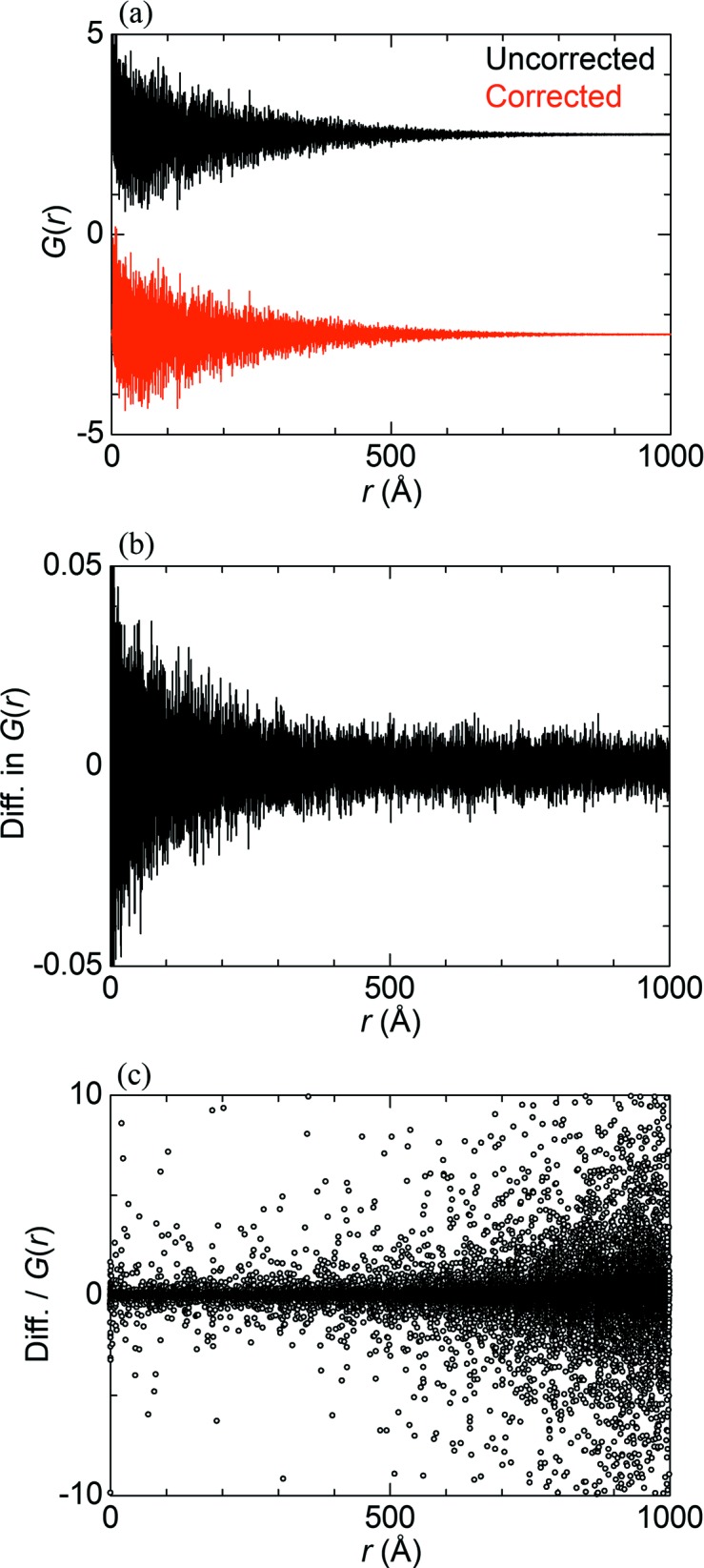
PDF *G*(*r*) of Ni powder up to 1000 Å in interatomic distances. Firstly, (*a*) is the *G*(*r*) based on the uncorrected and corrected data, which are shifted up and down, respectively. Secondly, (*b*) is the difference in *G*(*r*) between the uncorrected and corrected data and (*c*) is the ratio of the difference to the *G*(*r*).

Fig. 11 ▸ shows the PDF *G*(*r*) of Ni powder up to 1000 Å in interatomic distance *r*. The *G*(*r*) based on the uncorrected data appears to be similar to that based on the corrected data [Fig. 11 ▸(*a*)]. The amplitude of the *G*(*r*) is two orders of magnitude larger than that of the difference between the uncorrected and corrected PDFs [Fig. 11 ▸(*b*)]. On the other hand, the amplitude of the *G*(*r*) is damped towards the *r* corresponding to the crystallite size, whereas that of the difference is almost constant at *r* above 300 Å. Fig. 11 ▸(*c*) shows the ratio of the difference to *G*(*r*). The ratio is found to increase with increasing *r*. The results indicate that the systematic error caused by XRNU affects the PDF at higher *r* rather than at lower *r* since the amplitude of the PDF at higher *r* is much smaller than that at lower *r*.

## 4. Discussion   

The present study was intended to develop a statistical approach to correct XRNU in microstrip detectors. Indeed, the multi-step process combined with the divided-accumulation technique reduced the PNR from 11.6 to 2.3 at the intensity of 1.4 × 10^6^ photons, indicating an approach to the Poisson noise. As a result, a number of hidden Bragg reflections of Si powder, the intensity of which is four orders of magnitude lower than that of 111, were clearly observed. The diffuse scattering of SiO_2_ glass was obtained up to 27 Å^−1^ in *Q*. The resultant PDF of TiO_2_ nanoparticles was improved in a wide range of interatomic distances.

### 4.1. Origin of XRNU   

In contrast to the Poisson noise, the TFU of the uncorrected data of SiO_2_ glass reaches a plateau at intensities above 10^4^ photons, as shown in Fig. 6 ▸(*a*). The difference in TFU between the Poisson noise and the uncorrected data is estimated to be ∼0.8% at 10^6^ photons. In photon-counting detectors, the dispersion of a threshold energy is recognized as one of the most important factors for XRNU. The threshold dispersion in MYTHEN can be reduced by the threshold-equalization technique (trimming) using the internal 6-bit digital-to-analog converter by a factor of 15 (Bergamaschi *et al.*, 2010 ▸). Nevertheless, it has been reported that there still remains a dispersion of nearly 1% (Bergamaschi *et al.*, 2010 ▸), which is larger than the Poisson noise above 10^4^ photons. The remaining dispersion after the trimming is found to be close to the TFU in the uncorrected data. It follows from these arguments that most of the XRNU is caused by the threshold dispersion. Moreover, it is known that the distribution of the threshold energy is a function of ambient temperature as well as threshold energy. For this reason, the trimming for MYTHEN has been conducted at a fixed temperature for specific energies. In the present study, the same experimental booth temperature was maintained during measurements of correction factors and samples, minimizing the XRNU. In other words, correction factors must be collected every time the threshold energy and the ambient temperature are changed.

### 4.2. Alternative to the flat-field calibration   

The flat-field calibration, which has been conventionally used to correct XRNU in X-ray detectors, has been applied to MYTHEN (Bergamaschi *et al.*, 2010 ▸; Thompson *et al.*, 2011 ▸). The conventional calibration requires that a whole detector should be illuminated by uniform X-rays with a specific energy. We, however, have found it difficult to generate such X-rays to a precision of 0.1%, which corresponds to the Poisson noise at 10^6^ photons. Another problem is that the conventional calibration does not take into account the Poisson noise. Therefore, the large XRNU as observed at both ends of channels can be corrected (Thompson *et al.*, 2011 ▸), whereas the conventional calibration has failed in correcting the XRNU at intensities above 10^4^ photons. In contrast, the alternative approach is based on the statistical estimation that the scattering intensity at a fixed angle from an object is expected to be constant within the Poisson noise. The approach is also characterized by the use of any scattering object to correct XRNU in principle. We emphasize that our approach has succeeded in exploiting the dynamic range and the spatial resolution in MYTHEN for the first time.

### 4.3. XRNU correction for short-scale PDF   

Let us compare our experimental method with the RA-PDF method described in Section 1, where a flat-panel detector was employed, in terms of the reduced structure function *F*(*Q*) of SiO_2_ glass. In RA-PDF, the *F*(*Q*) at high *Q* has been reported to be improved by increasing the measurement time, leading to a *Q*_max_ of 35 Å^−1^ (Chupas *et al.*, 2007 ▸). Similarly, the *F*(*Q*) based on our corrected data is found to be improved according to photon statistics (Fig. 9 ▸). Area detectors such as the flat panel and the imaging plate can average out XRNU between pixels when two-dimensional data are converted into one-dimensional data for data analysis. Such an averaging effect cannot be expected in microstrip detectors such as MYTHEN. From these arguments, we emphasize that XRNU in microstrip detectors should be corrected in terms of short-scale PDF analysis as well as multi-scale analysis.

### 4.4. Need for the XRNU correction in MYTHEN   

MYTHEN has been employed as a detector for high-resolution and high-throughput powder diffraction experiments at synchrotron facilities (Bergamaschi *et al.*, 2010 ▸; Thompson *et al.*, 2011 ▸). Moreover, total-scattering measurements have been attempted taking advantage of its resolution (Haverkamp & Wallwork, 2009 ▸; Saleta *et al.*, 2017 ▸). Such attempts, however, have not been successful in obtaining the reliable PDF over a wide range of interatomic distances. Here let us compare the present study with the previous study in terms of the PDF analysis of anatase-TiO_2_ nanoparticles. In the previous study, the *R*_w_ for the distance range 1.5–20 Å was 26% (Haverkamp & Wallwork, 2009 ▸), which is larger than that of the present study by 10% [Fig. 10 ▸(*c*)]. The discrepancy in *R*_w_ between the two results may be attributed to not only the difference in *Q*_max_ but also the XRNU at high *Q*. In the present study, the *R*_w_ for the range 80–100 Å is improved by the XRNU correction by 27%, which is, however, still higher than that for the range 1.5–20 Å. This could be ascribed to non-uniformity in crystallite size and shape since the range is close to the crystallite size. In addition, the PDF analysis of micrometre crystal Ni clearly indicates that the impact of XRNU on PDF is significant with increasing interatomic distance [Fig. 11 ▸(*c*)]. Based on these findings, we emphasize that the XRNU in MYTHEN needs to be corrected to obtain the reliable PDF over a wide range of distances.

### 4.5. Stepwise updating of correction factors in the multi-step process   

As shown in Fig. 7 ▸, the single-step process takes about 80 h to reach 0.25% in TFU, whereas the multi-step process takes about 8 h to reach the same TFU. For the single-step, the correction factors *c*_ss_ in equation (2) were calculated based on 160 measurements, in which case 1280 channels were partitioned every eight channels. For the multi-step, although the partitioning in the fifth step was close to that of the single step, the correction factors *c*_ms_ in equation (13) were calculated based on only 24 measurements. The substantial reduction in hours and measurements can be interpreted by introducing global and local correction factors. In the first step of the multi-step, 16 measurements were performed by shifting the detector system by 80 channels to obtain the correction factors *c*1 for each channel, which can be defined as global correction factors for the second step. By using the global factors, only two measurements should be performed by shifting the detector system by half of 80 channels to obtain *c*2, which can be regarded as local correction factors for the second step. Note that there is no need to carry out 32 (= 1280/40) measurements by using global factors before calculating local factors. Next, *c*1 × *c*2 can be used as the global factors for the third step. Indeed, local correction factors *c*2 in equation (6), *c*3 in equation (8), *c*4 in equation (10) and *c*5 in equation (12) were obtained based on global factors *c*1, *c*1 × *c*2, *c*1 × *c*2 × *c*3 and *c*1 × *c*2 × *c*3 × *c*4, respectively. As a result, correction factors at each step are stepwise updated as shown in Fig. 3 ▸. That is the reason why the multi-step process can significantly save hours and measurements to correct XRNU.

### 4.6. Limitations of our approach   

We acknowledge that there are several limitations of our approach to correct XRNU. As shown in Fig. 6 ▸(*a*), there remains a discrepancy of ∼0.1% between the corrected data and the Poisson noise at 10^6^ photons. The discrepancy could be ascribed to some random errors, except for the statistical error which is impossible for the approach to correct. Fig. 6 ▸(*b*) shows the systematic discrepancy in PNR as a function of average intensity. These results imply that the random error such as the thermal noise, which is negligible at intensities below 10^5^ photons, becomes significant at intensities above 10^5^ photons. From this point of view, the mask in front of the sensor plays an important role in reducing part of the random error (Fig. 7 ▸). Based on these arguments, we conclude that our approach is powerful in correcting systematic errors such as XRNU.

In the present study, measurements for correction factors took as long as ∼80 h to evaluate the time evolution of the TFU. Compared with the typical measurement time for a sample, this time is too long. As shown in Fig. 7 ▸, however, the TFU reachs a plateau in about 30 h, indicating that one day would be sufficient thanks to the stepwise updating described in Section 4.5. Furthermore, correction factors need to be collected for each threshold energy since most of the XRNU in MYTHEN are considered to be caused by the dispersion of the threshold energy. In other words, once correction factors for a fixed threshold energy are obtained, these factors can always be applied to scattering data measured at the same energy.

## 5. Conclusion   

We have succeeded in developing an unbiased approach to correct the XRNU in microstrip detectors such as MYTHEN and evaluated the quality of total-scattering data in terms of the TFU and PNR, which are proposed as figures of merit for X-ray detectors in the present article, as well as *F*(*Q*) and PDFs. The principle of our approach could be applied to not only microstrip detectors but also pixel detectors such as PILATUS (DECTRIS Ltd). Unlike the conventional flat-field calibration, our alternative approach can minimize XRNU over a wide range of intensities, making best use of the dynamic range of the detectors. Consequently, the approach facilitates a discrimination between the systematic error, such as the XRNU, and the random error, such as the thermal and Poisson noise, leading to the development of new X-ray detectors with a PNR of 1. We believe that the present study provides a new benchmark for the evaluation of X-ray detectors and their data. In the near future, we will develop a data-driven approach to the PDF based on high-accuracy (PNR ≃ 2 at 10^6^ photons) and high-resolution (Δ*Q* ≃ 10^−4^ Å^−1^) total-scattering measurements over a wide range of *Q* (∼30 Å^−1^) to obtain 0.1 Å-resolution PDFs at interatomic distances above 100 Å, leading to the visualization of heterogeneous phenomena in functional nanomaterials.
